# 术者之行始于扶镜

**DOI:** 10.3779/j.issn.1009-3419.2016.06.14

**Published:** 2016-06-20

**Authors:** 辉 李

**Affiliations:** 100020 北京，首都医科大学附属北京朝阳医院胸外科 Department of Thoracic Surgery, Beijing ChaoYang Hospital, Capital Medical University, Beijing 100043, China

**Keywords:** 扶镜手, VATS, Camera-holder, VATS

## Abstract

胸外科手术技术已发生革命性变化，胸腔镜手术（video-assisted thoracic surgery, VATS）已经、正在成为各类胸部手术的主流方式。安全、高质、艺术化的VATS离不开扶镜手与术者默契的团队配合，如果说优秀的开放手术是术者脑、眼、手、体并用并无缝连接的整体配合的结果，那么，VATS的扶镜手则担负着两个人的眼睛，即本人和术者，这比照顾一个人脑、眼、手、体更为精细，更为困难。当然，优秀的扶镜手必将成长为不久将来优秀的术者。

## Abstract (en)

The techniques of thoracic surgery has undergone evolutionary changes, and currently video-assisted thoracic surgery (VATS) has already been or is going to be the predominant procedure of various thoracic surgeries. The safe and artistic VATS with high quality is closely associated with the cooperation of camera-holder and the surgeon. If an excellent thoracotomy is the result of perfect integral cooperation of the brain, eyes, hands and the body of the surgeon, the camera-holder in VATS procedure, then, is responsible for the eyes of both the surgeons and himself. This is more meticulous and difficult than that for a single person's brain, eyes, hands and body. Meanwhile, an excellent camera-holder will undoubtedly become an excellent surgeon in the foreseeable future.

## 1 目前在腔镜手术中扶镜存在的问题和误区

胸腔镜已经成为胸外科手术最重要的工具，被广泛应用于各类手术中。许多年轻医师对于术者有着强烈的追求，但往往忽视了胸腔镜手术（video-assisted thoracic surgery, VATS）手术中的另一个重要的角色-扶镜者的作用。

胸腔镜的优势是通过镜头位置及方向的调节，获得胸腔内各个角度清晰的视野，这种视野往往是开放手术所不能及的。正是有了这种视野，才使得胸外科医生能够进行各种精细、安全的手术操作。胸腔镜手术是一个团队工作，除了术者之外，扶镜手也非常重要。扶镜质量直接关系到手术的成败，因为在胸腔镜手术中术者要根据视觉反馈的信息进行手术操作，因此，胸腔镜就是术者的“眼睛”，扶镜者的作用就是要保证这只“眼睛”清晰、明亮、视物距离适当^[1, 2]^。目前已经比较流行的单孔胸腔镜手术则对扶镜手有了更高的要求。

但是当前现状就是很多胸外科医师对扶镜工作的重视程度不够，一方面表现在扶镜手往往是低年资医师，例如实习医师、低年资住院医师等等，这些医师由于对于手术理解不够、对具体步骤了解少，造成了在扶镜过程中存在许多不足，另一方面，缺乏相关的培训机制，往往是毫无经验就上岗。本文试图针对临床问题做有针对性的介绍。

## 2 扶镜的方法与技巧

### 2.1 了解镜头特性

扶镜者除了掌握胸腔镜的设备组成、工作原理和使用方法外，最重要的是要了解镜头的特性。

临床常用的胸腔镜有0°镜和30°镜两种，前者视野较窄，不能提供的角度视野。30°镜为广角视镜，可提供多角度视野，目前最常用。在使用30°胸腔镜之前，扶镜者应对其特性有所了解才能在术中根据术者操作的需求运用自如，满足手术需要。镜头进入胸腔后扶镜者对于胸腔镜的操作主要集中在胸腔镜的底座及光纤上。胸腔镜底座平是得到正确术野的基础。

首先扶镜者要学会胸腔镜下观察空间和方向。最基本的动作就是把镜头摆正，即光纤朝上，这时看到的图像实际上是俯视角度，通常用来显示正常解剖位置，如胸壁在上、肺在下。在此基础上，通过调节光纤的方向来改变观察目标的方位，使胸腔镜实现对目标的立体观察。例如30°胸腔镜光纤调至正下方时就得到前视和仰视角度；当光纤左偏或右偏则分别得到向右或向左的侧视角度。只有灵活运用了30°镜头的这些特性，才能实现多角度观察。

光纤的旋转功能一般用于以下几方面：例如对血管的游离，需要从血管的不同侧面进行观察，充分打开血鞘，以达到血管的裸化。还有就是当镜头方向与术者器械方向相同时，要想观察到器械头端的工作情况避免副损伤就必须适当的旋转光纤。再就是在手术开始放置戳卡及手术结束后检查戳卡孔时，为了确切观察到有无戳卡造成的脏器损伤或戳卡穿刺造成的胸壁出血，必须将光纤进行旋转。

## 3 进镜前准备

要想做一个合格的扶镜者，在扶镜前做好充分的思想准备是必要的，因为扶镜是一项非常艰苦和单调的工作，需要长时间注意力集中和保持手臂抬起的固定姿势，费脑、费眼、费力。

胸腔镜从普通镜头到高清镜头的发展向我们体现了术者对清晰术野的追求。清晰的术野不仅可以让术者心情愉悦，更能增强术者的视觉分辨能力，减少术中并发症的发生。想要得到一个较为持久的清晰视野有很多影响因素，但术前对镜头的处理显得尤为重要。由于体腔与室温的差别，腔镜进入人体后常会出现水雾，严重影响视野。为此，人们尝试了很多办法如：碘酒擦拭镜头、防雾水镜头等，效果均不理想。目前最简单和最有效的方法是60 ℃-70 ℃热水浸泡镜头，并且整个术程中用来浸泡镜头的水温都应保持在60 ℃左右。第一次浸泡镜头时间要稍长，约1 min左右，好让镜头充分预热，高于胸腔内温度，之后每次镜头模糊后浸泡时间可以仅为几秒即可。镜头浸泡结束后就需要擦拭，擦拭物可选择柔软的无菌纱布。擦镜的顺序为先镜身后镜面，擦拭镜面时要稍用力，反复擦拭2遍-3遍，务必使镜面无残留的水滴及水雾。整个擦镜动作要尽可能迅速，让镜头尽快进入胸腔，避免镜头温度冷却，这样就可以使镜头不易起雾，较长时间维持一个清晰的术野。

此外，进镜前要调整好焦距和白平衡以确保图像清晰、色彩还原逼真。

## 4 术中扶镜技巧

### 4.1 保持镜头稳定，保证术野在镜头中央

扶镜者持镜必须稳定，防止晃动、抖动和旋转。移动镜头时要平稳缓慢，不要像电影中的远距离推拉。作为胸腔镜手术来说，将术者需要观察的目标置于显示器中央时就能构成一个和谐的画面。任何对于显示器角落或边缘目标的观察都会让人觉得难受，觉得对目标的观察不全面，手术操作不确切、不安全。

### 4.2 适时进退

扶镜者应根据术者操作的精细程度来调整视野的纵深。保持胸腔镜底座平、观察目标正中是对术野的水平调整，而胸腔镜的进退则是对术野纵深的调整。随着胸腔镜越靠近观察目标，目标将被进一步放大，对细节操作的观察越清晰。根据胸腔镜与手术靶区的距离，术野的纵深可分为近距、中距和远距。近距视野用于显示细微结构，如对血管的裸化（打开血管鞘）或清扫上纵隔淋巴结时。中距视野有利于观察局部的详细情况，适用于较精细的操作，如肺动脉的游离。远距视野开阔，有利于了解术野的整体感，了解病变位置及其与周围的关系。例如，在对术野进行大的调整时需要使胸腔镜远离术野，这样观察范围扩大，便于术者与助手在直视下同时调整，增加调整的准确度，缩短调整时间。手术结束清理术野时，远距术野可以加强术者对操作范围的整体把握，避免术野活动性出血等情况的遗漏。但注意过近会使胸腔镜的焦距无法调整清晰。

### 4.3 注意力高度集中，与术者保持默契

扶镜者是术者的眼睛，眼睛是由大脑支配的，手术台上的大脑只有一个，就是术者的大脑，无论扶镜者还是助手都必须想术者之所想，时时和术者保持一致，这样的配合才默契，手术才流畅。要做到默契，首先扶镜者要熟悉手术过程和步骤，其次注意力要高度集中。一个好的扶镜者，往往可以做到有预见性，即让镜头向术者下一个术野移动，这对增加术者动作的连贯性。因此，扶镜者不能只做到术者做哪看哪，每一次的术野转换都需要术者亲自调整镜头方向。当然，每个术者的手术习惯不同，因此，相对固定的团队组合是必要的，而无固定搭配很难训练出好的扶镜者。

### 4.4 确保镜头清晰

术中有很多情况会使镜头污染而变得模糊，例如脏器的接触、液体喷溅、电刀或超声刀产生的热气、套管内壁的血迹等等，因此确保镜头的清晰是扶镜者非常重要的工作。扶镜者可以通过一些方法来减少或避免镜头被污染，如定时用纱布或棉签清理套管内壁；术者进行电刀或超声刀操作时，将镜头后撤或偏向一侧；进镜时速度要慢，防止脏器碰脏镜头。在这里需要强调的时，如果术中镜头被污染需要擦拭，一定要告知术者并获得同意，这样术者会停止操作，不要自作主张随意撤镜，以免术者在操作过程中突然失去视野导致误伤。还有就是当出现较大出血时，千万不要擅作决定撤出镜头，虽然时间很短，但可能导致术者失去对出血的有效控制。正确的处理时当遇到大出血特别是喷射性时，应及时移动镜头方向，避开血液喷射的方向，使术者用最有效的方法先控制出血，然后再决定止血方法。

## 5 小结

胸腔镜手术是一个团队工作，扶镜者的重要性是每个胸腔镜手术医生都能深刻体会到的，但很少胸腔镜医生重视对扶镜者的培养。在我们看来，胸腔镜手术的扶镜有很多技巧，只有在反复的训练实践中不断的总结才能掌握和提高。同时，我们也应建立相关的培训课程，加强对扶镜基本功和技巧的培训。唯有这样，才能充分发挥胸腔镜的视觉优势，帮助术者安全流畅的完成每一个胸腔镜手术，扶镜者从这一刻开始也逐步向术者行列迈近^[3]^。
